# Efficient ilamycins production utilizing *Enteromorpha prolifera* by metabolically engineered *Streptomyces atratus*

**DOI:** 10.1186/s13068-023-02398-w

**Published:** 2023-10-05

**Authors:** Yu-Xi Jiang, Gao-Fan Zheng, Long-Chao Chen, Na Yang, Xiu-Juan Xin, Jun-Ying Ma, Jian-Hua Ju, Hui Wu, Ming Zhao, Ruida Wang, Fa-Liang An

**Affiliations:** 1 State Key Laboratory of Bioreactor Engineering, School of Biotechnology, East China University of Science and Technology, 130 Meilong Road, Shanghai, 200237 China; 2 Department of Applied Biology, School of Biotechnology, East China University of Science and Technology, 130 Meilong Road, Shanghai, 200237 China; 3 CAS Key Laboratory of Tropical Marine Bio-Resources and Ecology, Guangdong Key Laboratory of Marine Materia Medica, RNAM Center for Marine Microbiology, South China Sea Institute of Oceanology, Chinese Academy of Sciences, Guangzhou, 528225 China; 4 Marine Biomedical Science and Technology Innovation Platform of Lin-Gang Special Area, No.4, Lane 218, Haiji Sixth Road, Shanghai, 201306 China; 5 Anhui Engineering Laboratory for Industrial Microbiology Molecular Breeding, College of Biology and Food Engineering, Anhui Polytechnic University, Wuhu, 241000 China

**Keywords:** Ilamycins, Anti-tuberculosis, *Streptomyces atratus* SCSIO ZH16, *Entromorpha prolifera*, BldD

## Abstract

With the invasion of green tides and the increase of urban green areas worldwide, multimillion tons of *Enteromorpha* need to be reutilized. In this study, *Enteromorpha prolifera* powder is considered a promising biomass resource for the production of commercial chemical products production. Ilamycins, novel cyclic heptapeptides with significant anti-TB activities, are isolated from *Streptomyces atratus* SCSIO ZH16, a deep-sea-derived strain. Using EP powder as a nitrogen source, the production of ilamycins reached 709.97 mg/L through optimization of the nitrogen source using the engineered strain *S. atratus* SCSIO ZH16 Δ*R*. After mutant strain constructions and tests, strain *S. atratus* SCSIO ZH16 Δ*R*::*bldD* EP powder achieved a higher production titer of ilamycins. Furthermore, the production titer of ilamycins and ilamycin E reached 1561.77 mg/L and 745.44 mg/L, respectively, in a 5 L bioreactor. This study suggests that *E. prolifera* is a promising and eco-friendly nitrogen source for the production of ilamycins.

**Supplementary Information:**

The online version contains supplementary material available at 10.1186/s13068-023-02398-w.

## Introduction

In the past 10 years, severe eutrophication of seawater has resulted in the occurrence of *Enteromorpha prolifera* (EP) green tides, which are harmful algal blooms causing significant damage to the economy and environment of coastal cities in Asia [22]. Due to its high organic matter content and abundant production, however, EP has the potential to be used as raw materials for digestion [13]. Carbohydrates in EP exist in the polymer forms of hexose and pentose, which can be subsequently converted into bioethanol or biohydrogen [3]. EP proteins serve as a source of N-containing valuable products and nutrients for humans or animals. They can also be hydrolyzed into amino acids and subsequently converted into commercial chemical products [11]. Therefore, the efficient reutilization and disposal of EP as a source of energy and materials can help mitigating greenhouse gas emissions. However, only a few studies have been reported on the application of EP [11]. Actinomycetes species are renowned for the capacity to produce a multitude of high-valued product, even using waste biomass [5, 12]. Previous studies have demonstrated that *S*. *atratus* SCSIO ZH16, isolated from deep-sea sediment in the South China Sea, exhibits advantages, including rapid growth, ease of cultivation, and stable metabolism, for the production of high-value products [19, 20]. Among the natural products from *S*. *atratus* SCSIO ZH16, nonribosomal peptides ilamycins, especially ilamycin E, show brilliant anti-tuberculosis properties [9], and require yield improvement for the realization of industrial production or process. By Plackett–Burman design model, single factor optimization, pH coordinated feeding and continuous pulse feeding, [7] achieved 415.7 ± 29.2 mg/L ilamycin E yield in a 300 L bioreactor. Our previous research also improved the production of ilamycin E from 13.51 to 762.50 mg/L in a 5 L bioreactor, and found the crucial role of nitrogen source on cellular growth of ZH16-Δ*ilaR* and the production of ilamycins [24]. Soybean powder, the preferable nitrogen source identified, has obvious shortcomings, such as difficult to sterilize and high cost. Therefore, it is crucial to develop an efficient production process that can utilize inexpensive nitrogen sources to achieve massive production of ilamycin E.

Beside optimization of fermentation conditions, genetic modification is another powerful tool for the improvement of antibiotic-producing strains [2]. Commonly used targets in *Streptomyces* genetic modification include metabolism pathways that supply or shunt the precursors, resistance proteins that protect the host, modifying factors that determine the activity of enzymes, and regulators that promote or repress secondary metabolism, etc. [6]. Among them, genetic operation towards regulatory genes is a promising approach. For example, global regulator BldD dominates the process of morphology and secondary metabolism in multiple *Actinomyces*, and overexpression of *bldD* has boosted the production of daptomycin, avermectin and moenomycin [4, 10, 17]. Therefore, this study combined both fermentation condition optimization and strain genetic modification to enhance the yield of ilamycins.

By utilizing EP powder *as a cost-effective, environment friendly* nitrogen source, the engineered strain *S*. *atratus* SCSIO ZH16 Δ*ilaR*::*bldD* with extreme environment tolerance achieved a production of 1561.77 mg/L of ilamycins and 745.44 mg/L of ilamycin E in a 5 L bioreactor.

## Results and discussion

### Nitrogen sources screenings for the production of ilamycins

Nutrients so called nitrogen sources are required for forming nitrogenous cell components or metabolites, selection of which is essential for fermentation optimization. Due to the specific impact of any kind of organic nitrogen source on the growth of bacteria and synthesis of natural products, one bacterium or its genetic mutant has unique preference for organic nitrogen sources. Previous studies indicated the significant effect of the selection of organic nitrogen source on the yield of ilamycins in *S. atratus* SCSIO ZH16 Δ*ilaR* [7, 24]. In view of this, this study started with the systematic screening of composite nitrogen sources on *S*. *atratus* SCSIO ZH16 Δ*ilaR* (Δ*R* for short), an engineering strain that accumulates highly anti-tuberculosis activity component ilamycin F and ilamycin *E*_1_/*E*_2_, for the production of ilamycins.

**Fig. 1:**
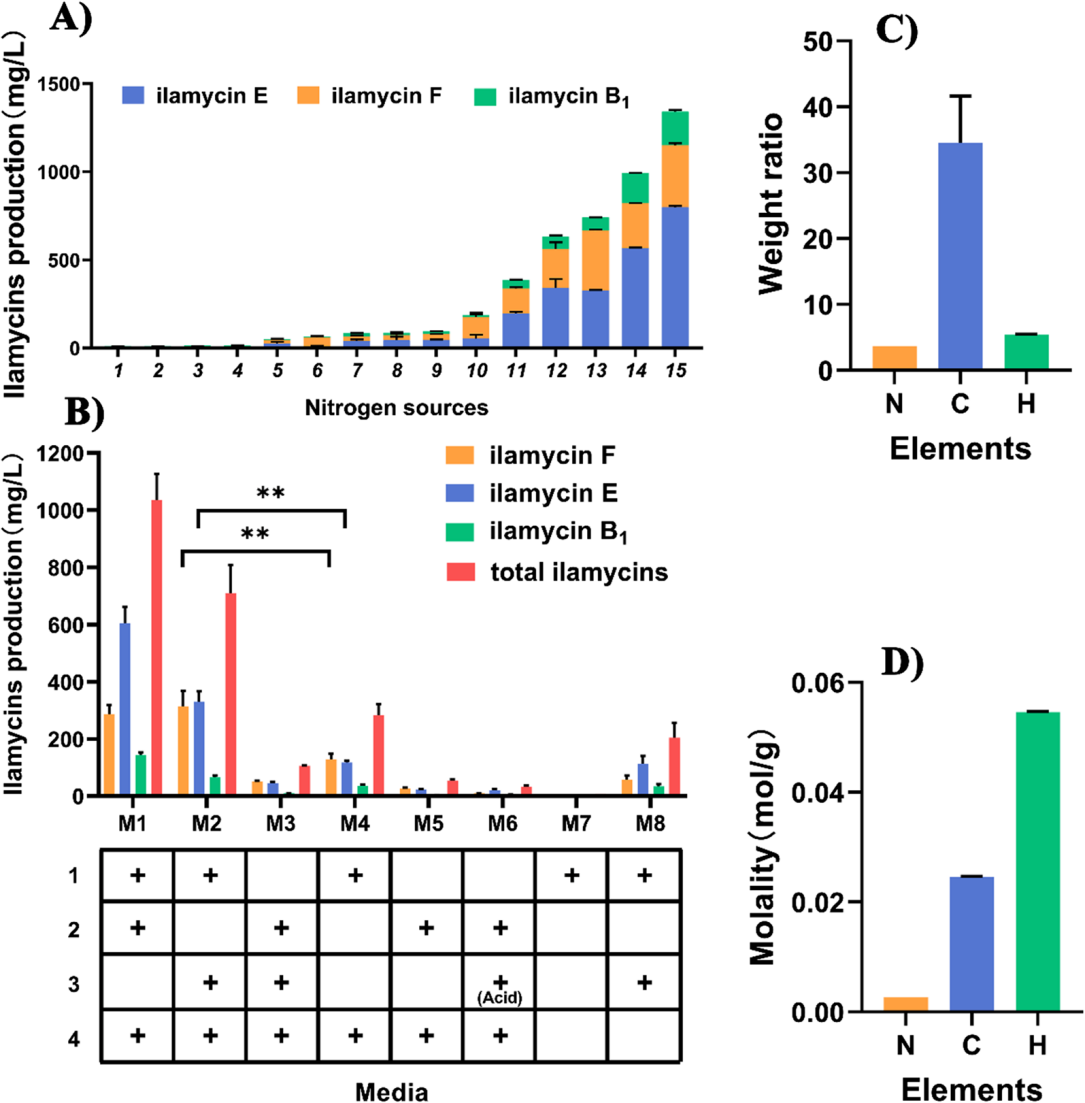
Utilization of EP powder for the ilamycins production in *S*. *atratus* SCSIO ZH16 Δ*R.* **A** Yield of ilamycins in media with different complex nitrogen sources. 1, Angel yeast extract powder; 2, acid-hydrolyzed casein; 3, tryptone; 4, corn steep liquor; 5, fish peptone; 6, bacterial peptone; 7, OXOID yeast extract; 8, yeast extract paste; 9, polypeptone; 10, peptone; 11, malt extract; 12, beef extract; 13, *Enteromorpha prolifera*; 14, bean flour; 15, soybean powder. **B** Effect of different combinations of carbon and nitrogen sources on the yield of ilamycins. 1, soluble starch; 2, soybean powder; 3, EP powder, ‘(Acid)’ means after acid hydrolysis; 4, corn steep liquor, NaNO_3_ and (NH_4_)_2_SO_4_. Error bars represent the standard deviations from three independent biological replicates. **: *P* < 0.01 (Student’s *t* test). **C** Weight ratio and **D** molality of N, **C**, **H** in EP powder

Given the shortcomings of soybean powder, currently used nitrogen source for ilamycins fermentation, such as difficult to sterilize, foaming properties, complicated production process and high cost, we attempted to seek a superior alternative. The 14 alternative nitrogen sources including EP powder, corn steep liquor, two types of yeast extract powder (Angel and OXOID), polypeptone, peptone, yeast extract paste, malt extract, beef extract, bacterial peptone, fish peptone, tryptone, acid-hydrolyzed casein and soybean flour were selected for screening. The results showed that soybean powder, bean flour and EP powder were the top three high-yielding nitrogen sources (Fig. 1A). Compared with the other two materials, EP powder has significant advantages in terms of environmental friendliness and cost.

To validate whether EP powder mainly function as the nitrogen source in fermentation, eight media varying in carbon and nitrogen sources (bottom of Fig. 1B, named M1–M8) were designed and tested. As shown in Fig. 1B, compared to M1 containing soybean powder, ilamycins yield of M2 containing EP powder decreased slightly (from 1044.97 to 709.97 mg/L), while M4 containing neither soybean powder nor EP powder produced only 117.73 mg/L ilamycins. Take the shortcomings of soybean powder into account, the yield reduction of replacing soybean powder with EP powder can be acceptable. In addition, when *S*. *atratus* was cultured in media containing both soybean powder (N source) and EP powder but lack of soluble starch (C source) (named M3 and M6), the production of ilamycins were extremely low, which means that EP powder cannot replace soluble starch as a carbon source, regardless of whether or not it has been subjected to acid hydrolysis. Besides, the ilamycins yield of M2 is 42.7-fold higher than that of original medium Am3 (Additional file 1: Fig. S1). These results demonstrate that selection of nitrogen source is crucial for the production of ilamycins, and EP powder can be used as a good nitrogen source substitute for cellular growth (Additional file 1: Fig. S2), primary and secondary metabolism flux regulation, and ilamycins biosynthesis of Δ*R*. In addition, macroalgal blooms result from eutrophication, and the consequent oxygen consumption, which threats the survival of other marine plants and animals while not release any toxic compounds [21]. Therefore, *E. prolifera* is non-toxic and can be utilized as sources of protein and bioactive compounds in formulated feeds, or processed into snacks and condiments [11]. Then, the results also showed that the yield in M8 definitely decreased compared to M2, which is main due to the absence of corn steep flour, because the trace element provided by corn steep liquor, including a variety of amino acids, inorganic salts and vitamins, are significant for the production of natural products [16]. What is more, the results of M5 and M7 add to the fact that the combination of the major organic nitrogen source EP powder, the carbon source soluble starch, the easy-utilized nitrogen sources corn steep flour, NaNO_3_ and (NH_4_)_2_SO_4_ are the most basic important requirements for production of ilamycins.

The analysis of element components on EP powder shows that the weight ratio of N, C and H elements are 3.69%, 29.58% and 5.46%, respectively, which converted to the molality ratio is 1:9:21 (Fig. 1C, D). The reason for the inability of EP powder to be utilized as a carbon source may due to that the main component of carbon source in EP powder is algal polysaccharides, which is not suitable for *Streptomyces*. In a word, when goes to industrial scale production, EP powder is a considerable environment friendly nitrogen source, and the leftover waste after product recovery may be treated as fertilizer or innocuous solid waste. As soluble starch is not considered as an ideal carbon source for industrial fermentation, we are currently attempting to screen alternative carbon sources and hope this issue would be solved in future.

### Construction of ilamycins high-yield strains

**Fig. 2:**
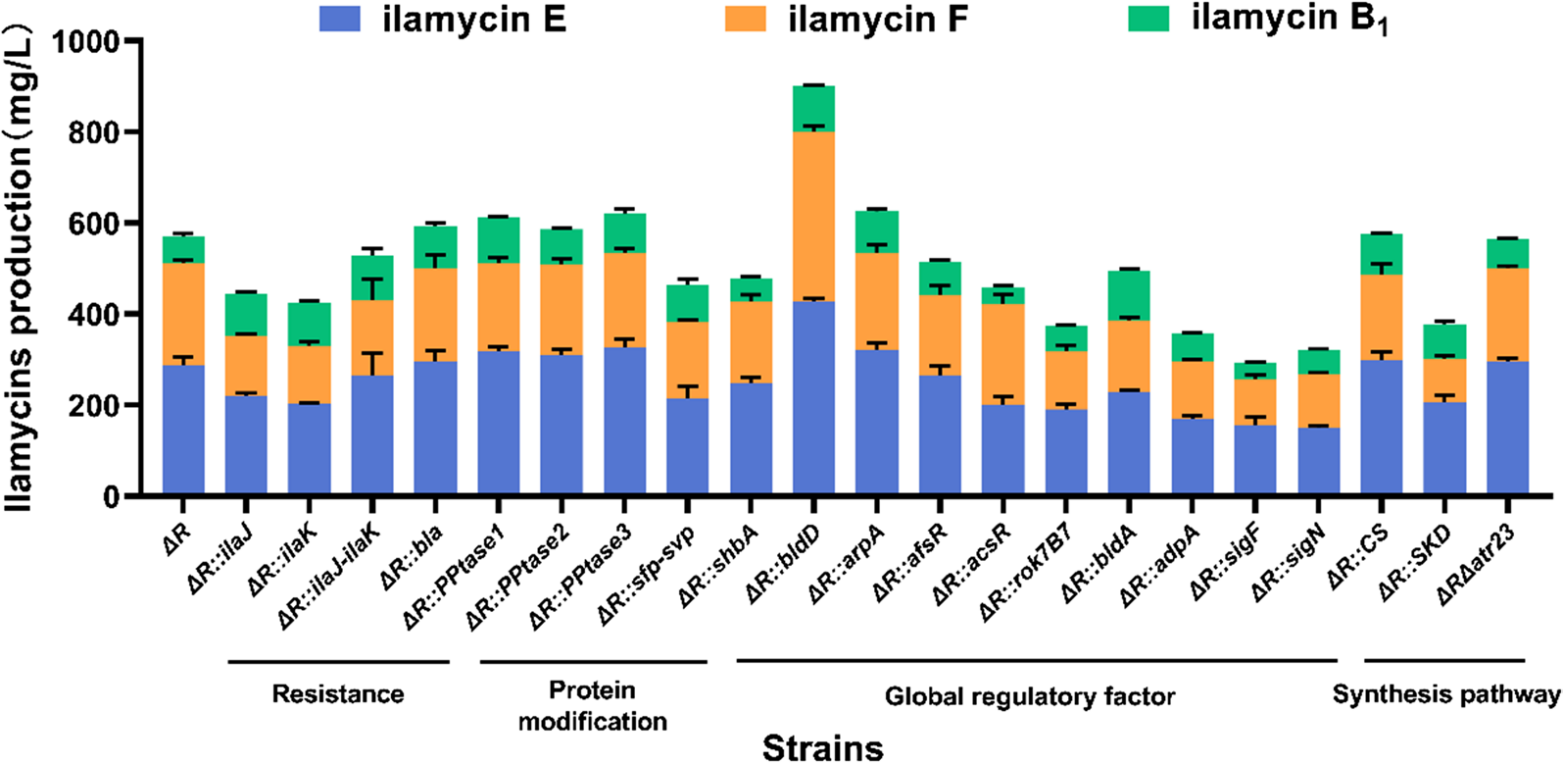
Ilamycins yields of engineered strains in EP powder culture medium after overexpression/knockout of candidate genes. Δ*R* is abbreviation of *S*. *atratus* SCSIO ZH16 Δ*R*; *Δ* and – denote knockout, overexpression, and co-expression, respectively

Given that the substitution of nitrogen source with EP powder caused decrease in ilamycins production to some extent, we turned to construct mutant stains that can increase ilamycins productions of *S*. *atratus* in EP powder-containing media EP powder. A screening on genes belonging to four function categories were performed:

1. *Self-resistance* Ilamycins resistance genes *ilaJ* and *ilaK* inside of the biosynthesis gene cluster and putative resistance gene *bla* (*RS37870*) were overexpressed by separately integrating them into the ΦC31 *attB* site. None of these overexpression strains led to enhancement in ilamycins production (Fig. 2). To be noticed, it has been found that overexpression of *ilaJ*, *ilaK* and *bla* promoted the production of ilamycin D and ilamycins *C*_1_/*C*_2_ in wild type (WT) strain (Additional file 1: Fig. S4), but these compounds have low anti-tuberculosis activity. Therefore, the cause of failure in ilamycins production improvement through *ilaJ*, *ilaK* and *bla* overexpression might be the specific transport towards ilamycin D and ilamycins *C*_1_/*C*_2_.
2. Biosynthesis pathway Two key enzymes from shikimate pathway, chorismate synthase (CS) and shikimate dehydrogenase (SKD), were selected for overexpression, since they participate in the supply of ilamycins biosynthesis precursor tryptophan and tyrosine. Beside precursor supply consolidation, *atr23* (encoding nonribosomal peptide synthetase) from the biosynthesis gene cluster of atratumycin was deleted by CRISPR–Cas9 to weaken the competitive pathway. Atratumycin is a decapeptide composed of amino acids, including tryptophan and tyrosine [14], its biosynthesis, therefore, theoretically inhibits the biosynthesis of ilamycins through competition of precursors. However, no enhancement of ilamycins yield occurred in strains Δ*R*::*CS*, Δ*R*::*SKD* and Δ*R*Δ*atr23* (Fig. 2). It can be speculated that the supply of tryptophan and tyrosine in strain Δ*R* is enough for ilamycins biosynthesis, and the shunt pathway for atratumycin biosynthesis is relative weak.
3. *Regulator* Manipulation towards regulator is a well-validated approach for yield enhancement of natural products [15, 23], and also adopted in this study. Well-known regulatory genes related to morphology development and secondary metabolism, such as *bldD*, *arpA* and *rok7B7 *etc., were overexpressed in strain Δ*R*, to explore the possibility of ilamycins yield increasement. Among the nine overexpression strains, Δ*R*::*bldD* showed an obvious enhancement in ilamycins yield by 58.13% to 900.04 mg/L (Fig. 2). It is noteworthy that, the effect evaluations of these regulatory genes were all carried out through gene overexpression because of the extremely low efficiency of CRISPR/Cas9 method in *S*. *atratus*. Further assessment on regulatory genes can be performed after optimization of the CRISPR/Cas9 method.
4. *Protein modification* As nonribosomal peptides, efficiently biosynthesis of ilamycins requires a protein modification executed by phosphopantetheinyl transferase (PPtase) to activate carrier proteins [1]. Therefore, overexpression of PPtase has successfully improved the production of multiple antibiotics [18]. In this study, two commonly used heterologous PPtase genes from *Bacillus subtilis* (*sfp*) and *Streptomyces verticillus* (*svp*), and three native PPtase genes (*RS01025*, *RS31655* and *RS38670*), were expressed in strain Δ*R*. None of the mutant strains based on PPtase genes led to enhancement of ilamycins yield (Fig. 2), indicating that the modification level of carrier proteins in strain Δ*R* is not a limiting factor for ilamycins biosynthesis.

In total, as the only high-yield strain screened, strain Δ*R*::*bldD* is used for the subsequent mechanism analysis and fermentation optimization.

### Mechanism analysis of BldD regulating ilamycins biosynthesis

**Fig. 3:**
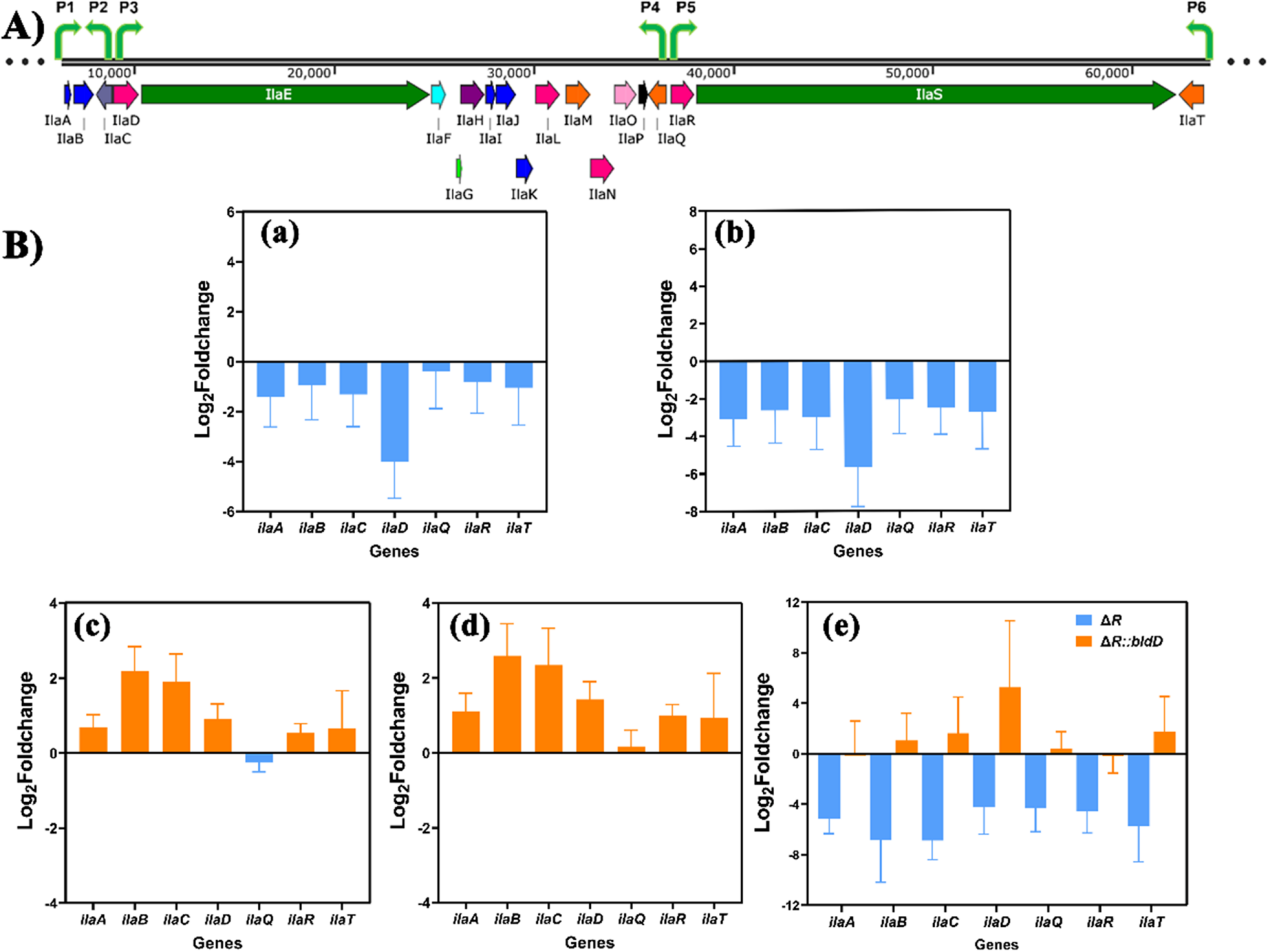
Effects of overexpressing *bldD* on transcription levels of ilamycins biosynthesis gene cluster at 48 h and 72 h. **A** Ilamycins biosynthesis gene cluster and predicted promoters P1–P6. **B** Transcription level changes of intra-cluster genes in Δ*R*::*bldD* compared to Δ*R*. (Two internal reference genes *hrdB* and 08190 were used). **a** 48 h, *hrdB* as internal reference gene; **b** 48 h, 08190 as internal reference gene; **c** 72 h, *hrdB* as internal reference gene; **d** 72 h, 08190 as internal reference gene; **e** fold changes of transcription levels of ilamycins biosynthesis gene cluster in strains Δ*R* and Δ*R*::*bldD* within 48 to 72 h. Error bars represent the standard deviations from three independent biological replicates

To understand the regulatory mechanism of BldD on the biosynthesis of ilamycins, the transcription level of biosynthetic genes of ilamycins were first examined. Genes inside ilamycins biosynthesis cluster form six operons: *ilaAB*, *ilaC*, *ilaDEFGHIJKLMNOP*, *ilaQ*, *ilaRS* and *ilaT* **(**Fig. 3A**)**, and the leading gene of each operon were selected for qRT-PCR detection. Since regulatory genes *ilaA* and *ilaB* have opposite functions, their transcriptional levels were both tested. RNA from strains Δ*R* and Δ*R*::*bldD* cultured after 48 and 72 h was extracted and detected by qRT-PCR. As shown in Fig. 3B, the transcription level of tested genes was decreased at 48 h, whereas up-regulated at 72 h. According to growth curve and ilamycins yield curve analyzed previously [24], the transformation of strain Δ*R* from primary to secondary metabolism occurs between 24 and 72 h. Therefore, we speculated that overexpression of *bldD* delayed the expression initiation of ilamycins biosynthetic genes at early stage while strengthened the expression level of them at mid-late stage. In addition, both the commonly used internal reference gene *hrdB* and the novel internal reference gene *RS08190* identified by our lab (encoding protein disulfide isomerase, unpublished data) were used in the qRT-PCR analyses. The almost identical results shown in Fig. 3B indicated that *RS08190* is transcript-stable and suitable for qRT-PCR analysis towards *S. atratus*.

Given the well-known role of BldD in morphology development, morphological observations on Δ*R*::*bldD* were performed. Compared to strain Δ*R*, formation of aerial mycelium and spores in strain Δ*R*::*bldD* on YMS medium is more prosperous, which was further proven by scanning electron microscopy (Additional file 1: Fig. S5).

### Optimization of fermentation conditions

**Fig. 4:**
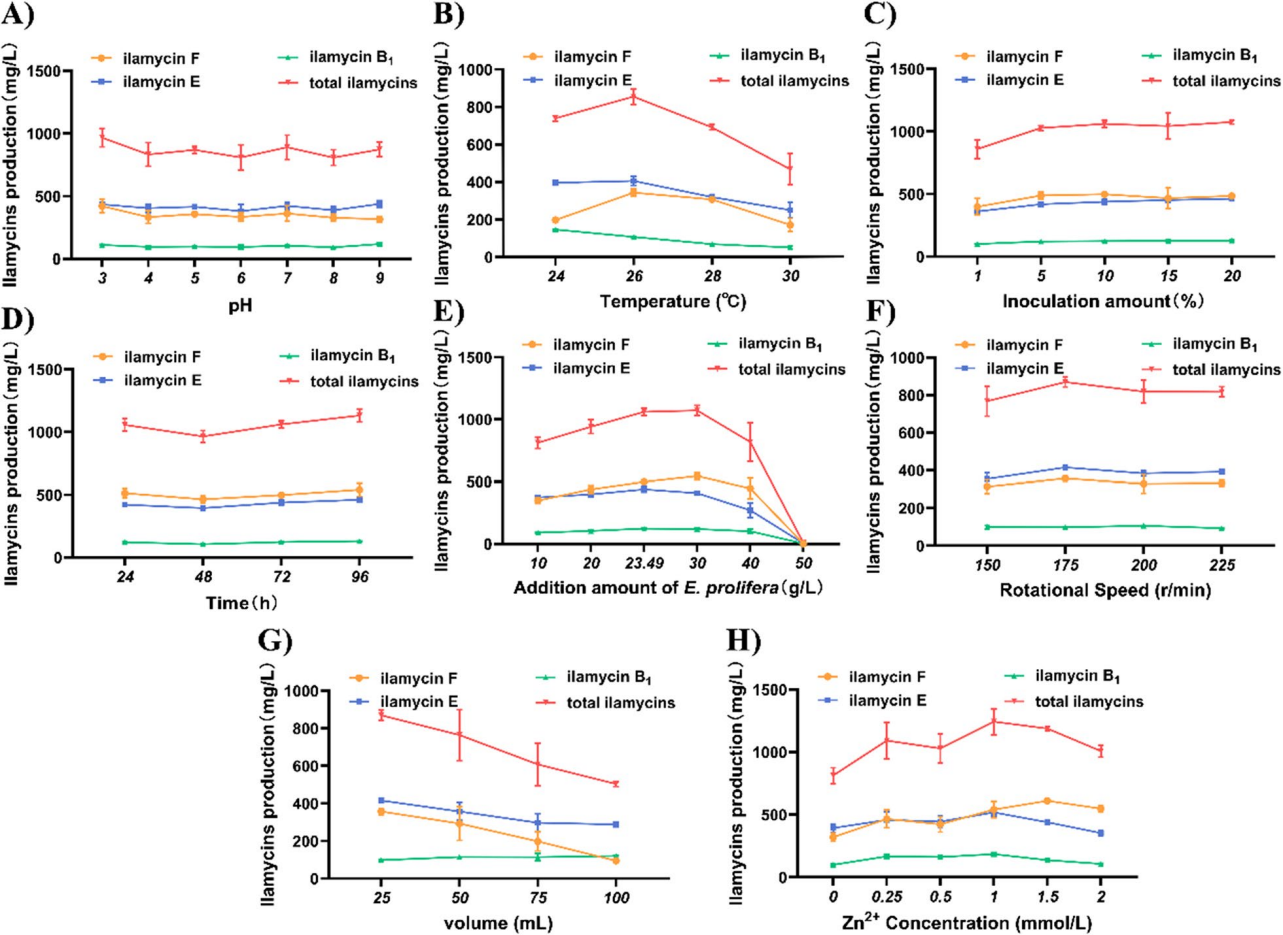
Effects of fermentation parameters in *E. prolifera* powder-containing media on the production of ilamycins by strain Δ*R*::*bldD*. **A** Initial pH; **B** temperature; **C** inoculation amounts; **D** inoculation time; **E** addition amount of Enteromorpha prolifera; **F** rotational speed; **G** liquid volume; **H** Zn^2+^ concentration. Error bars represent the standard deviations from three independent biological replicates

The fermentation process parameters are important factors affecting secondary metabolites. Thus, systematical optimization towards the key process parameters (pH, temperature, inoculation proportion, inoculation time, addition amount of EP powder, rotational speed, liquid volume and Zn^2+^ concentration) were performed on strain Δ*R*::*bldD*, to further improve the production efficiency of ilamycins (Fig. 4 and Additional file 1: Fig. S6).

After optimization, it was found that pH had no significant effect on the production of ilamycins. When pH = 3, the production of ilamycin E and total ilamycins were slightly higher. According to the investigation on the inoculum of seed liquid, 10% was the best inoculum proportion. As for key nitrogen source EP powder, 30 g/L was the best addition amount. To be noticed, excessive addition of EP powder will change the viscosity of the fermentation medium, thereby affecting the cellular growth and reducing the yield of ilamycins. Other process parameters (temperature, liquid volume, rotational speed, etc.) optimized were consistent with our previous work [24]. Another research in our lab found that addition of Zn^2+^ can further improve the yield of ilamycins, and we found in this work that 1 mmol/L is the best Zn^2+^ concentration for EP powder—containing medium.

To sum up, the optimal fermentation conditions of strain Δ*R*::*bldD* in shake-flask stage are as follows. The inoculation time is 96 h, the inoculation proportion is 10%, the initial pH = 3, the addition amounts of EP powder and Zn^2+^ are 30 g/L and 1 Mm, respectively, the liquid content is 25 mL (in 250 mL shake-flask), the fermentation temperature is 26 ℃, the rotational speed is 175 rpm, and the fermentation time is 216 h.

### Scale-up culture in a 5-L reactor

**Fig. 5:**
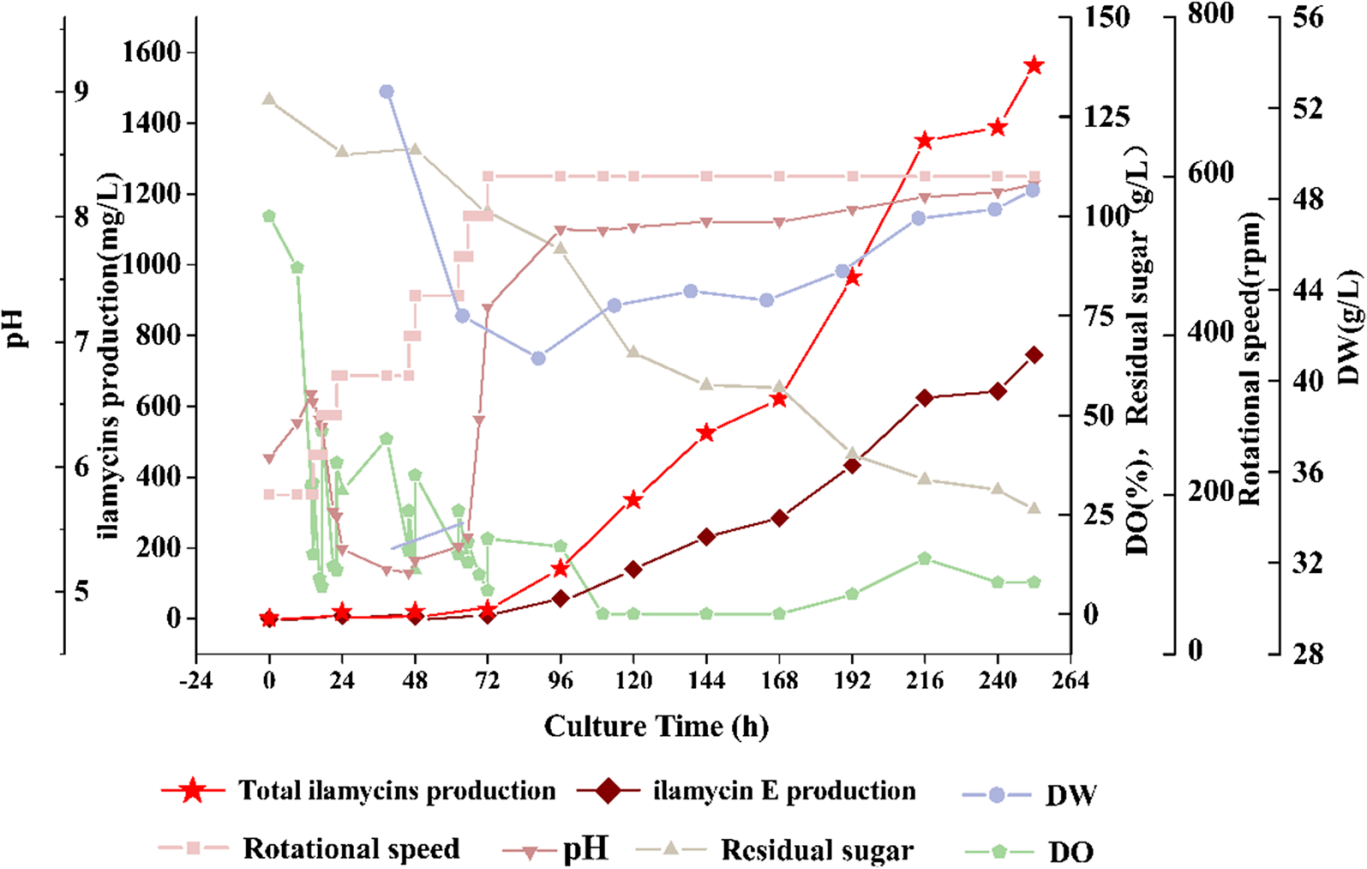
Time courses of ilamycins production, dissolved oxygen (DO), residual sugar, pH, rotational speed and dry weight (DW) during a 5-L fed-batch fermentation

Bioreactor fermentation is the vital step to achieve scale-up of the target molecule production, and the fermentation scale-up process from shake-flask level to 5 L reactor level was studied in this work (Fig. 5). The key parameters during the fermentation of strain Δ*R*::*bldD* were optimized as follows: the inoculation time is 96 h, the inoculation amount is 10%, the initial pH value is 3, the addition amount of EP powder is 30 g/L, the fermentation temperature is 26 ℃, the rotational speed is 175 rpm, the fermentation time is 216 h, the liquid content is 3 L. Production of ilamycin E in a 5 L reactor continued to rise and reached a peak of 745.44 mg/L on the 10th day, which is better than that of shake-flask culture (449.46 mg/L).

### Impact of seawater on production

**Fig. 6:**
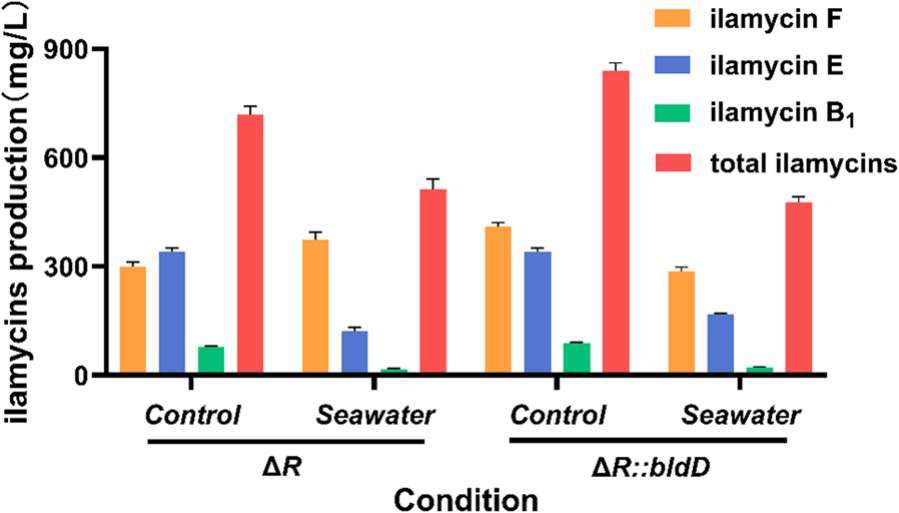
Ilamycins production of strains Δ*R* and Δ*R*::*bldD* in simulated seawater conditions. Error bars represent the standard deviations from three independent biological replicates

As a marine-origin algae, *E. prolifera* contains a large amount of sediment and metal ions from seawater before processing. To further reduce the amount of labor and cost, the feasibility of *S. atratus* strains to use fresh *E. prolifera* obtained from the sea directly was tested by adding artificial seawater into the medium. If the yields of ilamycins are not severely reduced, then simplification of the *E*. *prolifera* pretreatment may be considered. As shown in Fig. 6, both strains Δ*R* and Δ*R*::*bldD* can produce ilamycins after addition of artificial seawater, though the yields were decreased to some extent. This result indicates that *S*. *atratus* SCSIO ZH16 is capable of producing ilamycins under artificial seawater conditions, but we still need to consider both cost and yield comprehensively. Due to the difference between the seawater content in fresh *E. prolifera* and the above experiment, we may conduct more rigorous experiments in future to determine the most cost-effective solution for industrial production.

## Conclusions

In summary, waste biomass *E. prolifera* was utilized as a complex nitrogen source for the production of ilamycins in this work. The production-related genes of ilamycins were screened and engineered from the perspectives of antibiotic resistance, protein modification, regulatory factors and precursor supply. Fermentation results indicate that the *bldD* overexpression strain *S atratus* SCSIO ZH16 Δ*R*::*bldD* is more conducive to produce ilamycins using *E. prolifera*. Combining optimization of fermentation parameters and submerged fermentation, the production of ilamycins was increased to 1561.77 mg/L in a 5 L bioreactor. Therefore, the strategy of using EP powder to produce ilamycins is economy and high effectiveness, which meets the needs of industrial production and benefits for protecting the ecological environment.

## Materials and methods

### Strains, plasmids and primers

The bacterial strains and plasmids used in this study are listed in Table 1. All primers used in this study are listed in Additional file 1: Table S1 in Supplementary data, and synthesized by Azenta (Suzhou, China). *S. atratus* SCSIO ZH16 Δ*ilaR*, a genetically engineered mutant strain of *Streptomyces atratus* SCSIO ZH16 (NCBI Reference Sequence: NZ_CP027306.1), was given by Dr. Jianhua Ju’s laboratory (South China Sea Institute of Oceanology, Chinese Academy of Sciences, Guangzhou, China).

**Table 1:** Bacterial strains and plasmids used or constructed in this work

Strains and plasmids	Relevant phenotype	Source
Strains		
*E. coli*		
DH5α	*F-ϕ80lacZΔM15Δ(lacZYA-argF)U169recA1endA1hsdR17(rk-mk* +*)phoAsupE44λ-thi-1gyrA96relA1*	From Prof Wu
S17-1	*recA pro hsdR RP4-2-Tc::Mu-Km::Tn7*	From Prof Wu
*S. atratus*		
SCSIO ZH16	Wild type strain, ilamycins producer	From Prof Ma
Δ*R*	SCSIO ZH16 with in-frame deletion of *ilaR*	Lab stock
Δ*R*::*ilaJ*	Δ*R* Φ31 *attB*::pIBilaJ	This study
Δ*R*::*ilaK*	Δ*R* Φ31 *attB*::pIBilaK	This study
Δ*R*::*ilaJ*-*ilaK*	Δ*R* Φ31 *attB*::pIBilaJK	This study
Δ*R*::*bla*	Δ*R* Φ31 *attB*::pIBbla	This study
Δ*R*::*PPtase1*	Δ*R* Φ31 *attB*::pIBPPtase1	This study
Δ*R*::*PPtase2*	Δ*R* Φ31 *attB*::pIBPPtase2	This study
Δ*R*::*PPtase3*	Δ*R* Φ31 *attB*::pIBPPtase3	This study
Δ*R*::*sfp-svp*	Δ*R* Φ31 *attB*::pWHU2449	This study
Δ*R*::*shbA*	Δ*R* Φ31 *attB*::pIBshbA	This study
Δ*R*::*bldD*	Δ*R* Φ31 *attB*::pIBbldD	This study
Δ*R*::*arpA*	Δ*R* Φ31 *attB*::pIBarpA	This study
Δ*R*::*afsR*	Δ*R* Φ31 *attB*::pIBafsR	This study
Δ*R*::*rok7B7*	Δ*R* Φ31 *attB*::pIBrok7B7	This study
Δ*R*::*bldA*	Δ*R* Φ31 *attB*::pIBbldA	This study
Δ*R*::*adpA*	Δ*R* Φ31 *attB*::pIBadpA	This study
Δ*R*::*sigF*	Δ*R* Φ31 *attB*::pIBsigF	This study
Δ*R*::*sigN*	Δ*R* Φ31 *attB*::pIBsigN	This study
Δ*R*::*ilaQ*	Δ*R* Φ31 *attB*::pIBilaQ	This study
Δ*R*::*CS*	Δ*R* Φ31 *attB*::pIBCS	This study
Δ*R*::*SKD*	Δ*R* Φ31 *attB*::pIBSKD	This study
Δ*R*Δ*atra*	Δ*R* with in-frame deletion of *atr23(RS26165)* in BGC13	This study
Plasmids		
pKCcas9dO	A CRISPR/Cas9 editing plasmid harboring an *actII*-*ORF4*-specific gRNA and two homologous arms for in-frame deletion, *aac(3)IV*, *pSG5*	[1]
pKCcas9d*atr23*	A CRISPR/Cas9 editing plasmid harboring an *atr23(RS26165)*-specific gRNA and two homologous arms for in-frame deletion, *aac(3)IV*, *pSG5*	This study
pIB139	Integrative vector based on Φ31 *int*/*attP*, *aac(3)IV*, *oriT* RK2, PermE*	[2]
pIBilaJ	pIB139 harboring *ilaJ* under control of PermE*	This study
pIBilaK	pIB139 harboring *ilaK* under control of PermE*	This study
pIBilaJK	pIB139 harboring *ilaJ-ilaK* under control of PermE*	This study
pIBbla	pIB139 harboring *RS37870* under control of PermE*	This study
pIBPPtase1	pIB139 harboring *RS01025* under control of PermE*	This study
pIBPPtase2	pIB139 harboring *RS31655* under control of PermE*	This study
pIBPPtase3	pIB139 harboring *RS38670* under control of PermE*	This study
pWHU2449	pIB139 harboring *sfp-svp* under control of PermE*	[3]
pIBshbA	pIB139 harboring *RS19455* under control of PermE*	This study
pIBbldD	pIB139 harboring *RS07905* under control of PermE*	This study
pIBarpA	pIB139 harboring *RS12555* under control of PermE*	This study
pIBafsR	pIB139 harboring *RS24115* under control of PermE*	This study
pIBacsR	pIB139 harboring *RS33465* under control of PermE*	This study
pIBrok7B7	pIB139 harboring *RS32300* under control of PermE*	This study
pIBbldA	pIB139 harboring *RS16910* under control of PermE*	This study
pIBadpA	pIB139 harboring *RS15155* under control of PermE*	This study
pIBsigF	pIB139 harboring *RS21525* under control of PermE*	This study
pIBsigN	pIB139 harboring *RS21520* under control of PermE*	This study
pIBilaQ	pIB139 harboring *ilaQ* under control of PermE*	This study
pIBCS	pIB139 harboring *RS07940* under control of PermE*	This study
pIBSKD	pIB139 harboring *RS07945* under control of PermE*	This study

*Distinguish the engineered promoter from the native promoter

### Standard DNA manipulation, construction of overexpression and knock-out plasmids

Clones of *S*. *atratus* SCSIO ZH16 Δ*ilaR* were cultured in YEME medium containing 5 g/L polypeptone, 3 g/L yeast extract, 3 g/L malt extract, 10 g/L glucose, 5 mM MgCl_2_ and a natural pH. Genomic DNA was extracted from the cultured clones using the salting-out method. For PCR amplification, 2 × Hieff Canace^®^ Plus PCR Master Mix (With Dye) from vectors using T4 DNA ligase (TransGen Biotech, Beijing, China) or the Hieff Clone^®^ Plus One Step Cloning Kit (Yeasen Biotechnology, Shanghai, China). Plasmid MiniPrep Kit (Yeasen Biotechnology, Shanghai, China) was used for plasmid extraction, and the EasyPure^®^ Quick Gel Extraction Kit (Yeasen Biotechnology, Shanghai, China), was used for DNA product purification. DNA sequencing was carried out by Azenta (Suzhou, China). To amplify the complete open reading frame (ORF) of the target gene, primer pair x-F/R was used to amplify the gene from the *S. atratus* genomic DNA. The resulting PCR product was then digested using *Nde*I and *Eco*RI enzymes (Thermo Fisher Scientific, USA). Subsequently, the digested fragment was ligated with the linear site-specific integrative vector pIB139, resulting in plasmid pIB139-x. In the case of the *afsR* gene, since its sequence contains an *Eco*RI digest site, the ORF of *afsR* was ligated into the linear vector using the Hieff Clone^®^ Plus One Step Cloning Kit (Yeasen Biotechnology, Shanghai, China).To delete the NRPS gene *atr23* from *S*. *atratus* SCSIO ZH16, a previously reported CRISPR–Cas9 system was employed [8]. The construction of the pKCcas9d*atr23* plasmid involved three steps: (1) amplification of upstream and downstream homologous arms of atr23: the upstream and downstream homologous arms of *atr23* were amplified from *S*. *atratus* SCSIO ZH16 genomic DNA using PCR. Two primer pairs, uarm-*atr23*-F/R and darm-*atr23*-F/R, were used for this amplification. (2) Ligation of *atr23*-targeted sgRNA: a specific N20 sequence was incorporated into the sgRNA targeting *atr23*, and it was ligated upstream of the upstream homologous arm using the primer pair sg-*atr23*/uarm-*atr23*-R. This ligation generated sg-up-arm. (3) In-fusion cloning of sg-up-arm and down-arm into pKCcas9dO: the sg-up-arm and downstream homologous arm were then ligated into the pKCcas9dO vector between the *Spe*I and *Hin*dIII restriction sites using in-fusion cloning. This step was performed using the in-fusion cloning kit (Yeasen Biotechnology, Shanghai, China).

### Genome editing procedure

The *E*. *coli* DH5α was used to construct and amplify vectors and the *E*. *coli* S17-1 was used to transfer the recombinant plasmids into *S*. *atratus* SCSIO ZH16 Δ*ilaR* by conjugation. The desired kanamycin-resistant overexpression conjugants were checked by PCR and sequencing with primer pair 152-F/R. The desired apramycin-resistant deletion mutants of *atr23* were checked by PCR and sequence with primer pair knock-atra-F/darm-atra-R, and the strain knocked-out accurately was named Δ*R*Δ*atr23*.

### Media and growth condition

*S*. *atratus* SCSIO ZH16 Δ*ilaR* and its derivatives were grown on YMS agar (4 g/L yeast extract, 10 g/L malt extract, 4 g/L soluble starch, 10 g/L oatmeal, 2 g/L CaCO_3_, 20 g/L agar, pH 7.2–7.4) and incubated at 28 °C for 5 days to harvest matured spores. Then, four agar tablets (5 mm × 5 mm) were transferred into a 250 mL shake flask containing 25 mL seed medium (Am2ab medium). Am2ab medium is composed of 20 g/L glucose, 2 g/L peptone, 2 g/L yeast extract, 5 g/L soybean powder, 0.5 g/L MgSO_4_·7 H_2_O, 0.5 g/L KH_2_PO_4_, 4 g/L NaCl, 2 g/L CaCO_3_ and 30 g/L sea salt, pH 7.2–7.4. After shaking at 200 rpm and 28 ℃ for 72 h, 2.5 mL of the preculture medium was seeded into a 250 mL shake flask containing 25 mL fermentation medium (soluble starch 120 g/L, soybean powder 23.49 g/L, corn steep liquor 2.4 g/L, NaCl 6 g/L, NaNO_3_ 9.6 g/L, KH_2_PO_4_ 0.24 g/L, (NH_4_)_2_SO_4_ 4.68 g/L, CaCO_3_ 17.67 g/L) at 175 rpm, 26 ℃ for 216 h.

The tested media are listed in Table 2. For nitrogen sources variation, soybean powder was substituted with different nitrogen sources (EP powder, corn steep liquor, yeast extract powder, polypeptone, peptone, yeast extract paste, malt extract, beef extract, bacterial peptone, fish peptone, tryptone, acid-hydrolyzed casein, bean flour). All the nutrients were investigated with same quality fraction. To test the influence of EP powder on ilamycins production, soluble starch and soybean powder were substituted with EP powder of various concentrations and different pre-processed separately. All common biological and chemical reagents were obtained from standard commercial sources.

**Table 2:** Components of mediums

Medium	Component (g/25 mL)
M1	3 g soluble starch, 0.5873 g soybean powder, 0.06 g corn steep liquor, 0.15 g NaCl, 0.24 g NaNO_3_, 0.006 g KH_2_PO_4_, 0.117 g (NH4)_2_SO_4_, 0.4418 g CaCO_3_
M2	3 g soluble starch, 0.5873 g *E*. *prolifera*, 0.06 g corn steep liquor, 0.15 g NaCl, 0.24 g NaNO_3_, 0.006 g KH_2_PO_4_, 0.117 g (NH_4_)_2_SO_4_, 0.4418 g CaCO_3_
M3	1 g *E*. *prolifera*, 0.5873 g soybean powder, 0.06 g corn steep liquor, 0.15 g NaCl, 0.24 g NaNO3, 0.006 g KH_2_PO_4_, 0.117 g (NH_4_)_2_SO_4_, 0.4418 g CaCO_3_
M4	3 g soluble starch, 0.06 g corn steep liquor, 0.15 g NaCl, 0.24 g NaNO_3_, 0.006 g KH_2_PO_4_, 0.117 g (NH_4_)_2_SO_4_, 0.4418 g CaCO_3_
M5	0.5873 g soybean powder, 0.06 g corn steep liquor, 0.15 g NaCl, 0.24 g NaNO_3_, 0.006 g KH_2_PO_4_, 0.117 g (NH_4_)_2_SO_4_, 0.4418 g CaCO_3_
M6	1 g Acid-hydrolyzed *E*. *prolifera*, 0.5873 g soybean powder, 0.06 g corn steep liquor, 0.15 g NaCl, 0.24 g NaNO_3_, 0.006 g KH_2_PO_4_, 0.117 g (NH_4_)_2_SO_4_, 0.4418 g CaCO_3_
M7	3 g soluble starch, 0.15 g NaCl, 0.006 g KH_2_PO_4_, 0.4418 g CaCO_3_
M8	3 g soluble starch, 0.5873 g *E*. *prolifera*, 0.15 g NaCl, 0.006 g KH_2_PO_4_, 0.4418 g CaCO3
Am3	0.375 g soluble starch, 0.375 g bacterial peptone, 0.125 g soybean powder, 0.375 g glycerin, 0.05 g CaCO_3_, 0.75 g sea salt

### RNA isolation and transcript quantification

To verify the transcriptional level of intra-cluster genes of ilamycin BGC, total RNAs were extracted at 48 h and 72 h from different mutants grown in fermentation medium, using BioFlux^®^ SimplyP Total RNA Extraction Kit (BIoer Technology, Hangzhou, China). Reverse transcription was obtained using Hifair^®^ III 1st Strand cDNA Synthesis SuperMix for qRT-PCR (gDNA digester plus) (Yeasen Biotechnology, Shanghai, China). Real-time RT-PCR was performed in 96-well plate using the CFX Connect real-time PCR detection system (Bio-Rad), and the reaction solutions were prepared with Hieff UNICON^®^ Universal Blue qRT-PCR SYBR Green Master Mix (Yeasen Biotechnology, Shanghai, China). Housekeeping gene *RS08190* was chosen as a loading control. The transcriptional levels were calculated with three replicates using 2^−ΔΔCt^ value. Sequences of primers used in this study are listed in Additional file 1: Table S1.

### Fermentation in a stirred-tank 5-L bioreactor

The optimized fermentation medium and parameters were conducted in a 5 L bioreactor (Shanghai Guoqiang Biochemical Engineering Equipment Co., Ltd., Shanghai, China), which was equipped with pH, temperature and pO2 probes. 300 mL of seed culture was incubated for 4 days and then inoculated into the stirred-tank bioreactor containing 2.7 L fermentation media. The aeration was kept at 1.0–2.0 vvm (air/culture volume/min) and the agitation speed was set from 200 to 600 rpm, linking with the dissolved oxygen level. During the fermentation process, broth was cultivated at 26 ℃ for 240 h and samples were taken every 24 h to detect ilamycins production, dry weight and residual sugar of the broth.

### Analytical methods of ilamycins

As the EP powder, soybean powder and calcium carbonate were insoluble in water, it is hard to detect the dry cell weight, dry weight of mixed non-soluble particles and biomass were used instead. The supernatant and solid substances were separated by vacuum filtration. After 24 h of drying in a 50 ℃ oven, the dry weight was calculated using subtraction method. For the extraction and quantitative analysis of ilamycins by HPLC, detailed steps were performed as described previously [24]. The dried extracts of the fermentation products were dissolved with accurate amount of methanol and centrifuged for 5 min at 10,000 rpm followed by filtration of 0.22 μm membranes. Reversed phase column Agilent ZORBAX Eclipse XDB-C18 (4.6 mm × 150 mm, 5 μm) with UV detection at 210, 280, 354 nm was used to detect ilamycins under the following program: solvent system (solvent A, water supplemented with 0.1% acetic acid; solvent B, acetonitrile); 15% B to 71% B (linear gradient, 0–10 min), 71% B to 85% B (linear gradient, 10.0–11.5 min), 85% B (11.5–20.0 min), 85% B to 90% B (linear gradient, 20.0–20.1 min), 90% B (20.1–25.1 min), 90% B to 15% B (linear gradient, 25.1–26.0 min), 15% B (26.0–30.0 min); the flow rate was set as 1 mL/min.

The concentration of residual sugar in the broth were determined by a Total Carbohydrate Content Assay Kit (Solarbio Science & Technology Co., Ltd., Beijing, China). The content of carbon and nitrogen elements in the *E*. *prolifera* was detected through an Elemental analyzer (ELEMENTAR, German).

### Scanning electron microscope (SEM)

The *S. atratus* mutant strains were cultured on YMS medium at 28 ℃, and a 2 mm × 2 mm area of the strains was harvested from the plate at 3, 4, 5, 6, 7, 8 and 9 d by scalpel. The samples were soaked with 2.5% glutaraldehyde at 4 ℃ overnight and then dried naturally. Finally, the observation of the dehydrate cells were conducted on a S3400-N scanning electron microscopy (Hitachi, Tokyo, Japan) according to standard procedures.

### Supplementary Information

**Additional file 1: Fig. S1.** Comparison of ilamycins production of Δ*R* strain in M2 medium and Am3 medium. **Fig. S2.** Effect of different combinations of carbon and nitrogen sources on the dry weight of Δ*R* strain. **Fig. S3.** Reducing sugar, total sugar, nitrogen and oil content in EP powder before and after sterilization. **Fig. S4.** Effect of overexpressing *ilaJ* and *ilaK* on the production of ilamycins in wild type strain. **Fig. S5.** Macroscopic and microscopic morphological changes of strains Δ*R* and Δ*R*::*bldD*. **Fig. S6.** Effects of **A** pH, **B** temperature, **C** inoculation amount, **D** inoculation time, **E** addition amount of EP powder, **F** rotational speed, **G** liquid volume and (H) Zn^2+^ concentration on dry weight of Δ*R*::*bldD *strain fermentation broth. **Table S1 **Primers used in this study.

## Data Availability

Data will be made available on request.
