# Supplementary material for: Efficient ilamycins production utilizing Enteromorpha prolifera by metabolically engineered Streptomyces atratus
Source: Biotechnol Biofuels Bioprod. 2023 Oct 5;16:151. doi: 10.1186/s13068-023-02398-w (PMC10552367; doi:10.1186/s13068-023-02398-w)
Supplement: Supplementary file 1 — Additional file 1: Fig. S1. Comparison of ilamycins production of ΔR strain in M2 medium and Am3 medium. Fig. S2. Effect of different combinations of carbon and nitrogen sources on the dry weight of ΔR strain. Fig. S3. Reducing sugar, total sugar, nitrogen and oil content in EP powder before and after sterilization. Fig. S4. Effect of overexpressing ilaJ and ilaK on the production of ilamycins in wild type strain. Fig. S5. Macroscopic and microscopic morphological changes of strains ΔR and ΔR::bldD. Fig. S6. Effects of A pH, B temperature, C inoculation amount, D inoculation time, E addition amount of EP powder, F rotational speed, G liquid volume and (H) Zn2+ concentration on dry weight of ΔR::bldD strain fermentation broth. Table S1 Primers used in this study. [file 13068_2023_2398_MOESM1_ESM.doc]

**Additional file Materials**

**Efficient ilamycins production utilizing *Enteromorpha prolifera* by metabolically engineered** ***Streptomyces atratus***

Yu-Xi Jiang ^a, 1^, Gao-Fan Zheng ^a, 1^, Long-Chao Chen ^a^, Na Yang ^a^, Xiu-Juan Xin ^a^, Jun-Ying Ma ^c^, Jian-Hua Ju ^c^, Hui Wu ^a^, Ming Zhao ^e^, Ruida Wang ^a, b^*, Fa-Liang An ^a, d^*

*^a^ State Key Laboratory of Bioreactor Engineering, School of Biotechnology, East China University of Science and Technology, 130 Meilong Road, Shanghai 200237, China*

*^b^ Department of Applied Biology, School of Biotechnology, East China University of Science and Technology, 130 Meilong Road, Shanghai 200237, China*

*^c^ CAS Key Laboratory of Tropical Marine Bio-Resources and Ecology, Guangdong Key Laboratory of Marine Materia Medica, RNAM Center for Marine Microbiology, South China Sea Institute of Oceanology, Chinese Academy of Sciences, Guangzhou 528225, China*

*^d^ Marine Biomedical Science and Technology Innovation Platform of Lin-gang Special Area, No.4, Lane 218, Haiji Sixth Road, Shanghai 201306, China*

*^e^ Anhui Engineering Laboratory for Industrial Microbiology Molecular Breeding, College of Biology and Food Engineering, Anhui Polytechnic University, Wuhu 241000, China*

*Corresponding author:

[biord@mail.ecust.edu.cn](mailto:biord@mail.ecust.edu.cn) (R. Wang)

[flan2016@ecust.edu.cn](mailto:flan2016@ecust.edu.cn) (F.L. An)

^1^ These authors contributed equally to this work.

**Additional file materials**

**Fig. S1** Comparison of ilamycins production of Δ*R* strain in M2 medium and Am3 medium.

**Fig. S2** The effect of different combinations of carbon and nitrogen sources on the dry weight of Δ*R* strain.

**Fig. S3** Reducing sugar, total sugar, nitrogen and oil content in EP powder before and after sterilization.

**Fig. S4** Effect of overexpressing *ilaJ* and *ilaK* on the production of ilamycins in wild type strain.

**Fig. S5** Macroscopic and microscopic morphological changes of strains Δ*R* and Δ*R*::*bldD.*

**Fig. S6** Effects of (A) pH, (B) temperature, (C) inoculation amount, (D) inoculation time, (E) addition amount of EP powder, (F) rotational speed, (G) liquid volume and (H) Zn^2+^ concentration on dry weight of Δ*R*::*bldD* strain fermentation broth.

**Table S1** Primers used in this study.

**Fig. S1** Comparison of ilamycins production of Δ*R* strain in M2 medium and Am3 medium.


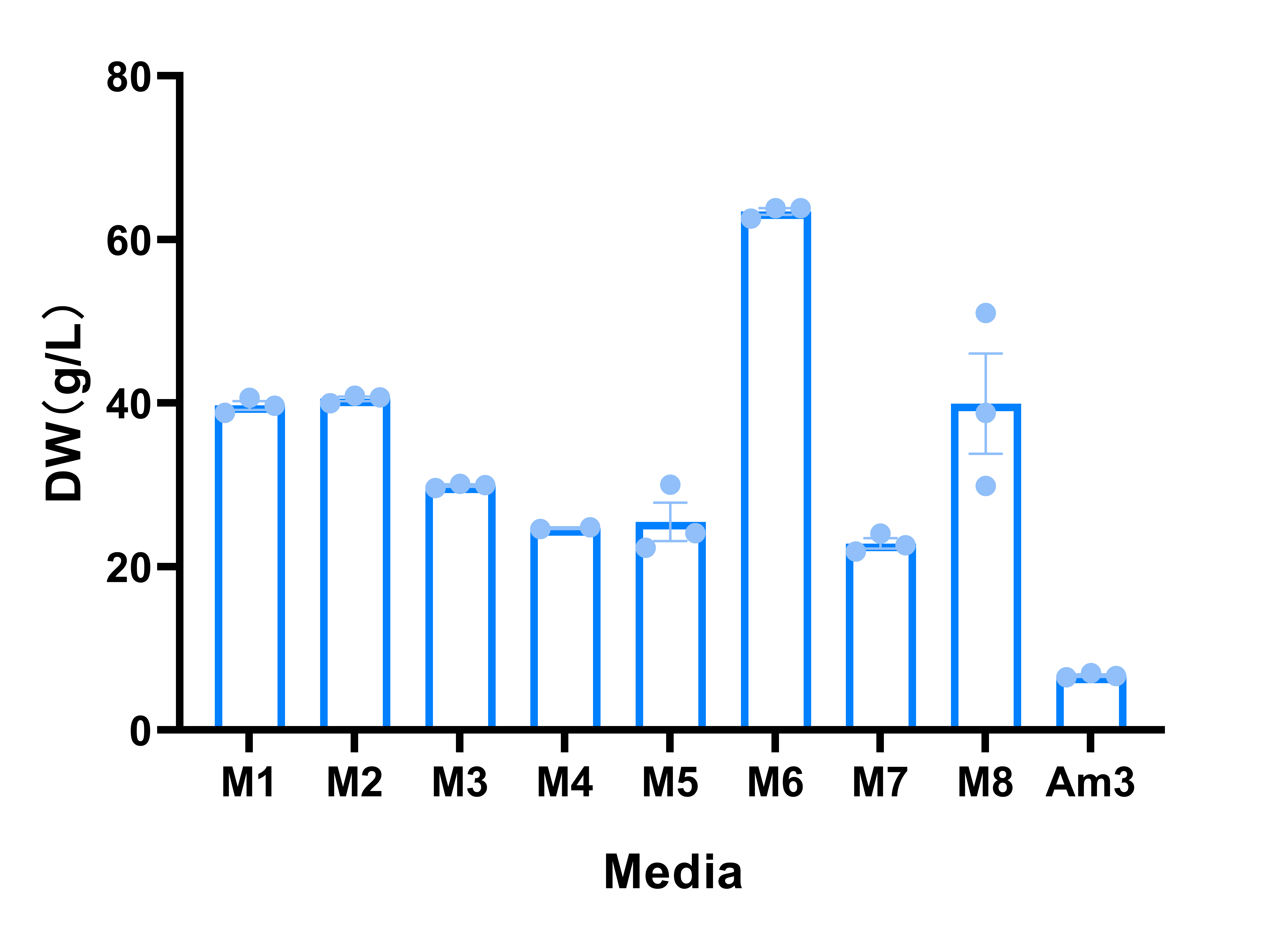


**Fig. S2** The effects of different combinations of carbon and nitrogen sources on the dry weight of Δ*R* strain


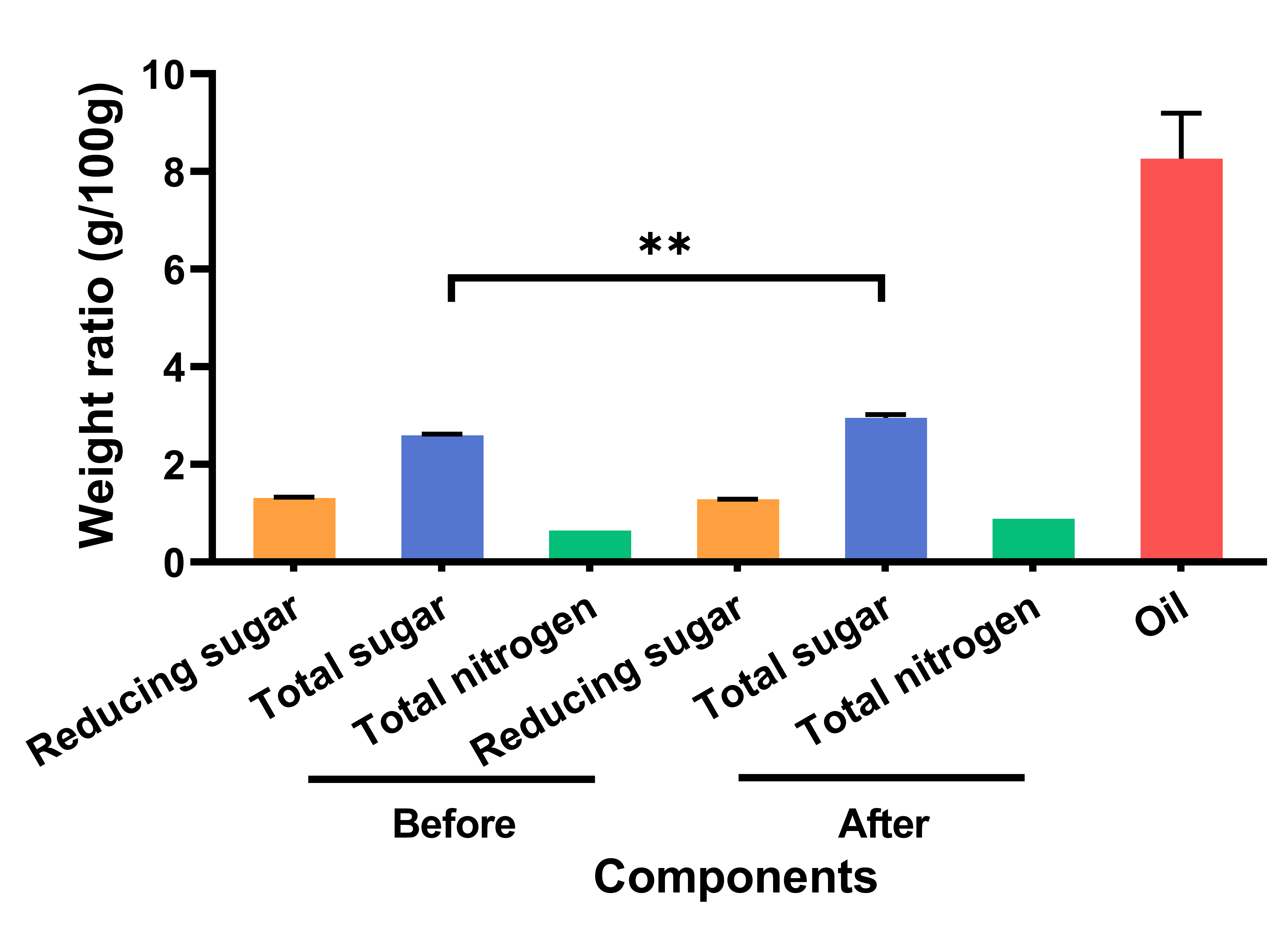


**Fig. S3** Reducing sugar, total sugar, nitrogen and oil content in EP powder before and after sterilization


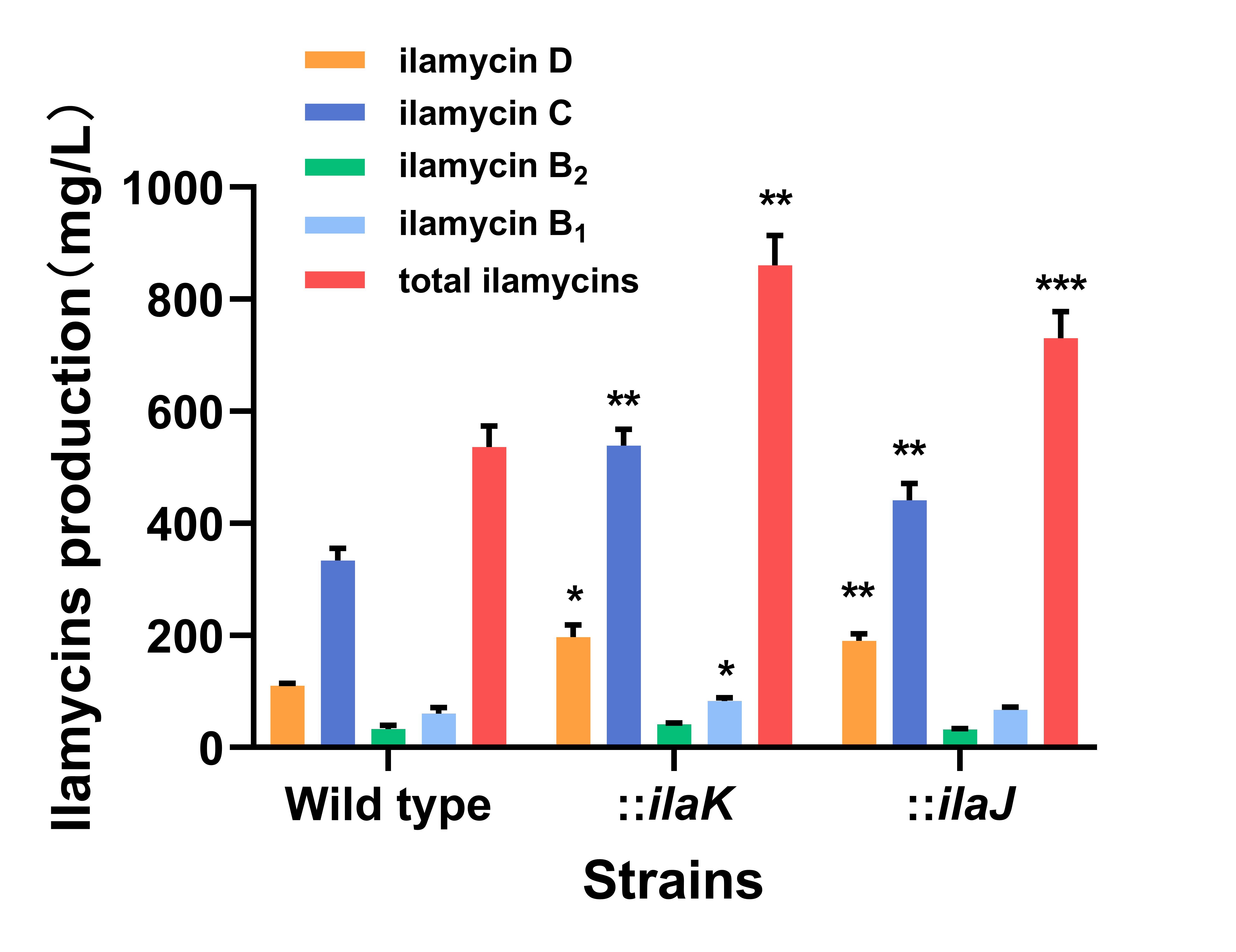


**Fig. S4** Effect of overexpressing *ilaJ* and *ilaK* on the production of ilamycins in wild type strain.

**
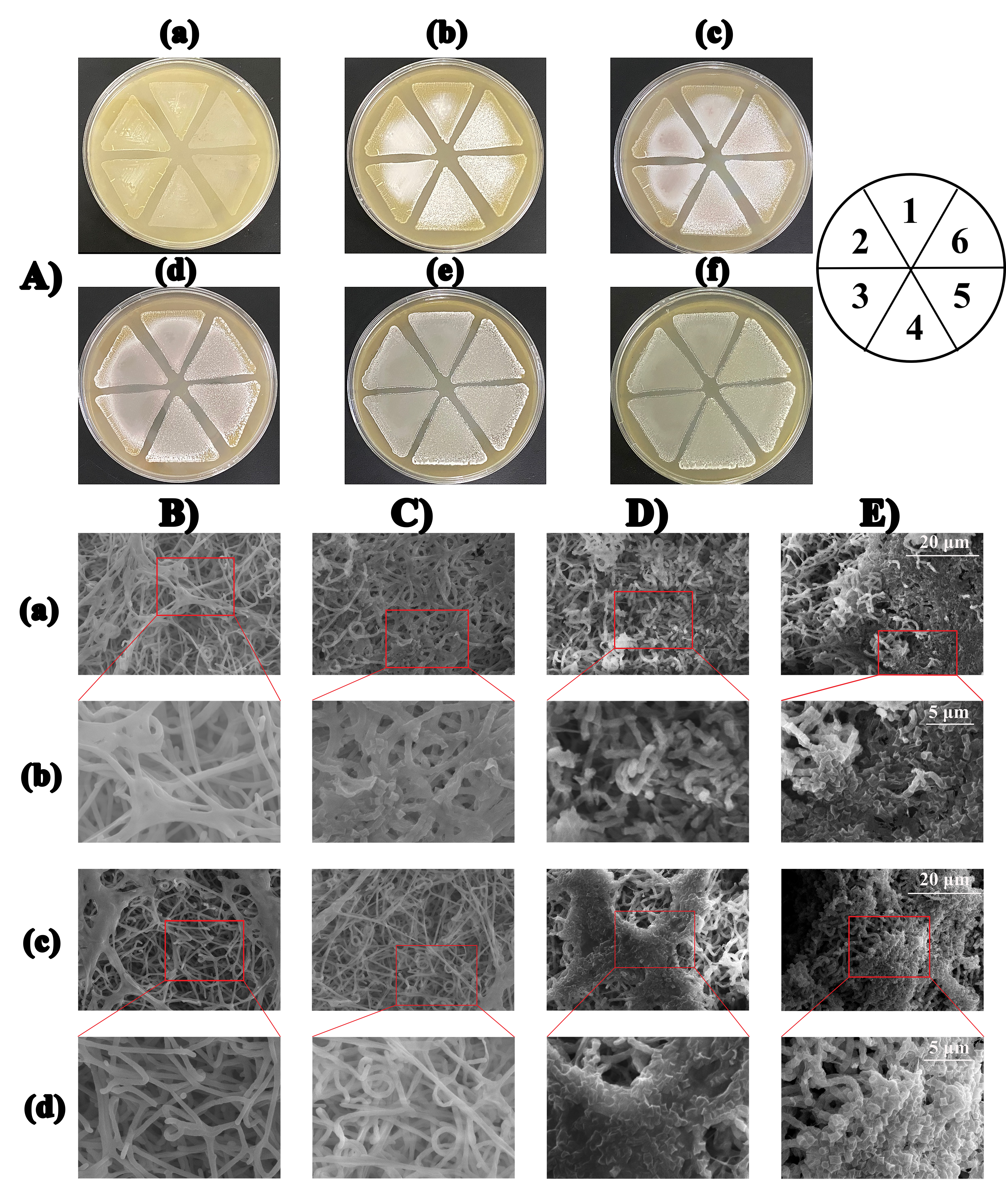
**

**Fig. S5** Macroscopic and microscopic morphological changes of strains Δ*R* and Δ*R*::*bldD.* (A) The macroscopic morphology of the bacteria referred to culture time of ‘3 d, 4 d, 5 d, 6 d, 7 d, 8 d’(corresponding to a-f, respectively), 1-3 being Δ*R*::*bldD*, 4-6 being Δ*R*. (B-E) Scanning electron microscopy was used to observe the morphological differentiation of strains Δ*R* and Δ*R*::*bldD* at 2300 and 6000 times on 4 d,5 d,7 d,8 d. (B) Day 4 (a-b) Δ*R*, (c-d) *bldD*; (C) Day 5 (a-b) Δ*R*, (c-d) *bldD*; (D) Day 7 (a-b) Δ*R*, (c-d) *bldD*; (E) Day 8 (a-b) Δ*R*, (c-d) *bldD*)


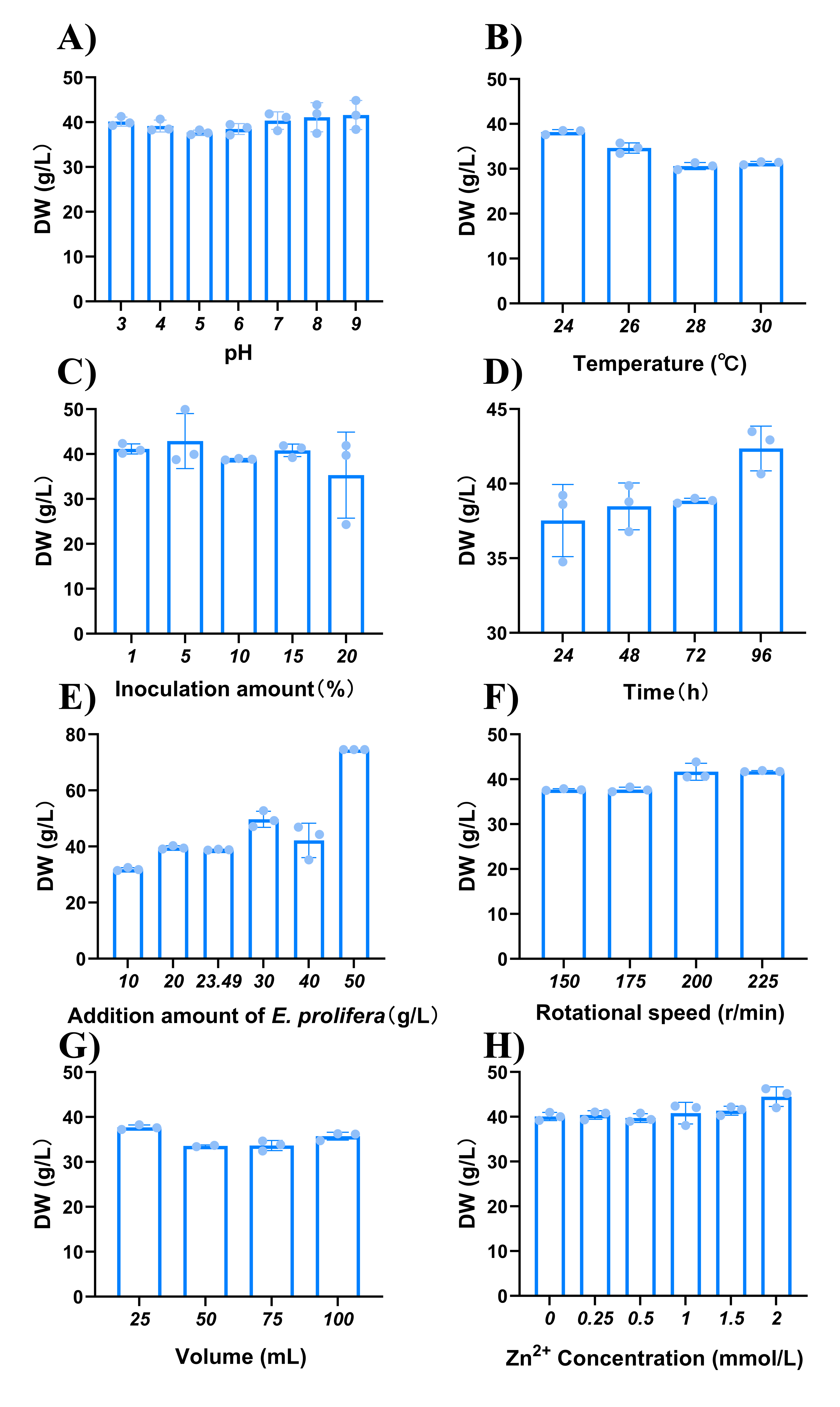


**Fig. S6** Effects of (A) pH, (B) temperature, (C) inoculation amount, (D) inoculation time, (E) addition amount of EP powder, (F) rotational speed, (G) liquid volume and (H) Zn^2+^ concentration on dry weight of Δ*R*::*bldD* strain fermentation broth.

**Table S1 Primers used in this study.**

| Primer | Sequence (5′–3′) |
| --- | --- |
| *PPtase1*-F | GATCCACATATGCCGCTGGTGATCACCGAG |
| *PPtase1*-R | TTACGAATTCTCAAAAAAAGGGAGGGCGGGT |
| *PPtase2*-F | GATCCACATATGATCGAGAAGATTCTGCCG |
| *PPtase2*-R | TTACGAATTCGCCAAGTACCGCAGCGGTG |
| *PPtase3*-F | GATCCACATATGACCGTGCTGTGGGGATCC |
| *PPtase3*-R | TTACGAATTCTCACCGAGCATGCCGCGG |
| *sigF*-F | GATCCACATATGCCGGCCAGTGCAGCGCCT |
| *sigF*-R | TTACGAATTCTTACGCCTCGATCCTGTTTG |
| *sigN*-F | GATCCACATATGTCCGCAGAACAGGGCAGC |
| *sigN*-R | TTACGAATTCTCAGTCGGCGATGAGCCCTTC |
| *shbA*-F | GATCCACATATGCCGGTGATTGCGCCCATG |
| *shbA*-R | TTACGAATTCTCATCCCTGCGCCTCCATCAC |
| *rok7B7*-F | GATCCACATATGGAGACTCCGGGGTCGCA |
| *rok7B7*-R | TTACGAATTCCTAAGTGAAGACAGGCGTGGCCG |
| *acsR*-F | GATCCACATATGCCGGAATCCGTCGATGCG |
| *acsR*-R | TTACGAATTCCGTCGCACTACGGCAGCAG |
| *bldD*-F | GATCCACATATGTCCAGCGAATACGCAAAAC |
| *bldD*-R | TTACGAATTCTCAGCCCTCTTCGTGGGC |
| *afsR*-F | **CCGGTTGGTAGGATCCACAT**ATGGACCGTGACGACCACG |
| *afsR*-R | **CTATGACATGATTACGAATT**TCAGGCGACTACGGCCGG |
| CX-*afsR*-1 | TTCAGCAAGGCGCTCGGCCT |
| CX-*afsR*-2 | CACCTGGTCGACCTGGACGT |
| CX-*afsR*-3 | CGACTGTCCTTGAAGACTTG |
| *arpA*-F | GATCCACATATGGCACAGCAGGCGCGC |
| *arpA*-R | TTACGAATTCTCAGTCGGGTGCGCCGGC |
| *ilaQ*-F | GGAATCCATATGACTCTTCCCGAGAATCG |
| *ilaQ*-R | CCGAGAATTCTCACTTCAGCAGTTCGACGA |
| *adpA*-F | GGAATTCCATATGATGAGCCAGGACTCCGCCG |
| *adpA*-R | CCGGAATTCCTACGGGGCACTCCGTTGAC |
| *bldA*-F | GGAATTCCATATGGCCCGGATGGTGGAATGCAG |
| *bldA*-R | CCGGAATTCTGGTGCCCGGAACCGGACTC |
| *ilaJ*-F | GATCCACATATGGAGACCGTCGGCGTGGTGAAGT |
| *ilaJ*-R | CCGGAATTCGGCGGTCGTCATCGGGTCTC |
| *ilaK*-F | GATCCACATATGACGACCGCCACGCGCAC |
| *ilaK*-R | CCGGAATTCTTATTCGCTCTTGTTGT |
| *ilaJK*-F | CCGCATATGAGGAGACCCGATGACGA |
| CX-JK-F | GTCATCGACCACGGAAAGCT |
| *bla*-F | GGAGCGCATATGGTGCTTGTAGGTGGCCTGG |
| *bla*-R | TTGCGAATTCTCAGTTGAGGGCGGCTGCCA |
| *sfp*-F | TCATATGAAAGGAGGCGGTGTCATGAAGATTTACGGAATTTATATGG |
| *sfp*-R | TGGATCCTTATAAAAGCTCTTCGTACGAGACC |
| *svp*-F | TGGATCCAAAGGAGGCGGTGTCGTGATCGCCGCCCTCCTGCC |
| *svp*-R | TGAATTCTTACGGGACGGCGGTCCGGTC |
| *CS*-F | GATCCACATATGAGCAGGTTGCGCTGGCTG |
| *CS*-R | TTCAGAATTCTCATCGGATCTGGAGGTGGTC |
| *SKD*-F | GATCCACATATGGCCTCGACTGACGGACGCC |
| *SKD*-R | TTACGAATTCTCAGCGTGCGGCCAGCGCCT |
| 152-F | GCGTAAGGAGAAAATACCGCATCAG |
| 152-R | TTCTGTGGATAACCGTATTACCGCC |
| sg-*atr23* | CAGTCCTAGGTATAATACTAG*AGGGGCGACCACAGGCTTAT*GTTTTAGAGCTAGAAATAGCAAGTTAAAATAAGGCTAGTCCGTTATC |
| uarm-*atr23*-F | TAGCAAGTTAAAATAAGGCTAGTCCGTTATCAACTTGAAAAAGTGGCACCGAGTCGGTGCACCCGGGTGATCAACTCCTC |
| uarm-*atr23*-R | CCTGGACGGACTCACCAACT |
| darm-atr*23*-F | AGTTGGTGAGTCCGTCCAGGGAAGGACAGGTCGAACTTGGAGA |
| darm-*atr23*-R | ACGACGGCCAGTGCCAAGCTCAGCTGTCCGGGCAGTTGGA |
| CX-*atr23*-F | GGTCCAGCTTCCCGTTGGAG |
| JD-*atr23*-F | GCTCGCCGATGACAGCTC |
| CR1 | GGTGCAAGCCCGGACGTT |
| CR2 | CCATTCAGGCTGCGCAACT |
| *q08190-*F | GATGCCGAGGAGATGGACAC |
| *q08190*-R | CGCTCGTCACCGATCACATA |
| *qhrdb*-F | TGAAGCAGATCGGCAAGGTTC |
| *qhrdb*-R | GATGATCTCCAGTTCGCGCTT |
| q*ilaA*-F | CTGGACGCGGATATTCTGCC |
| q*ilaA*-R | GATGGGATTTCACCCATGCTTT |
| q*ilaB*-F | TGAACAGCGAGCACGGACT |
| q*ilaB*-R | CAGTCGAACGCTGCCGTATC |
| q*ilaC*-F | GCGGTCTCGTCGAACATCTC |
| q*ilaC*-R | CCCTCGGACATGAAGGCGAT |
| q*ilaD*-F | CTGGTTTCAGAGAAGAGGCGAG |
| q*ilaD*-R | GTAGTCGCTCACCAACCACAT |
| q*ilaQ*-F | CGAAGGTGCTCACCCGAATC |
| q*ilaQ*-R | GAATGGGCGGAAAAAGCCC |
| q*ilaR*-F | GGCTTCTCTCCTTCGAGGTC |
| q*ilaR*-R | GCTGAAGAGGTCGTCCTTCG |
| q*ilaT*-F | CATGCCATTGCGCACATCC |
| q*ilaT*-R | CTGGTTCCAGCTGTACCTGTG |

^a^ underlining, restriction enzyme cutting site; **bold**, sequences homologous to pIB139; *Italics*, N20 of sgRNA.
